# An overview of point-of-care ultrasound for soft tissue and musculoskeletal applications in the emergency department

**DOI:** 10.1186/s40560-016-0173-0

**Published:** 2016-08-15

**Authors:** Kuo-Chih Chen, Aming Chor-Ming Lin, Chee-Fah Chong, Tzong-Luen Wang

**Affiliations:** 1 Emergency Department, Shin-Kong Wu Ho-Su Memorial Hospital, No.95 Wen-Chang Road, Shih-Lin District, Taipei City, 111 Taiwan, Republic of China; 2 School of Medicine, Fu-Jen Catholic University, New Taipei City, Taiwan

**Keywords:** Point-of-care, Ultrasound, Soft tissue, Musculoskeletal, Emergency medicine

## Abstract

**Background:**

The skin, soft tissue, and most parts of the musculoskeletal system are relatively superficial anatomical structures and ideal targets for ultrasound examination in the emergency departments. Soft tissue and musculoskeletal ultrasound applications are relatively underused compared to traditional emergency applications, such as trauma, abdominal aortic aneurysm, and chest and cardiovascular systems.

**Main text:**

It is important to have knowledge about sonoanatomy and landmarks within the skin, soft tissue, and musculoskeletal systems. Portable machines equipped with high-resolution transducers are now available to fulfill this field of applications in many emergency departments. After needling practice, emergency physicians can not only diagnose and identify pathological findings but also provide interventional procedures and treatments. In this review, we will introduce point-of-care ultrasound (POCUS) applications regarding the soft tissue and musculoskeletal systems: soft tissue infections, joint effusions, foreign bodies, long bone fractures, muscle and tendon injuries, vascular occlusions, and procedures.

**Conclusions:**

With POCUS, emergency physicians can visualize the structures beneath the skin and provide better and safer cares in the emergency departments.

**Electronic supplementary material:**

The online version of this article (doi:10.1186/s40560-016-0173-0) contains supplementary material, which is available to authorized users.

## Background

During the previous 20 years, many emergency physicians (EPs) are using focused ultrasound at bedside to manage challenging problems while providing cares to emergent and critically ill patients. Nearly 10 years ago, soft tissue and musculoskeletal applications were listed as one of core emergency ultrasound applications [1]. During the previous decade, most EPs are not familiar with soft tissue and musculoskeletal applications compared to other core applications, such as trauma, abdominal aortic aneurysm, deep venous thrombosis, central venous access, hydronephrosis, pneumothorax, and intrauterine pregnancy [2, 3].

The skin is the largest organ covering all the surface of the human body. The soft tissue and musculoskeletal structures beneath the skin are relatively superficial structures compared to visceral organs. Hence, the soft tissue and musculoskeletal systems should be easily observed under sonographic examination.

In this review article, we will introduce how to use point-of-care ultrasound (POCUS) for soft tissue and musculoskeletal applications in the emergency departments.

## Settings

**Fig. 1:**
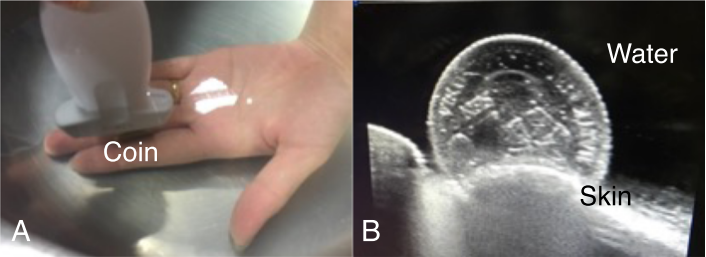
Water bath technique. The operator used a linear transducer in a water tank to scan a coin (**a**) and demonstrate a clear sonographic image of the coin surface (**b**)

A linear array transducer with a wide range of frequency is suitable for most scanning purposes. A curvilinear transducer can be used to scan deeper structures. Several methods can be used to create windows for better superficial examination, such as a copious gel, a commercial pad, a plastic intravenous bag, and a glove filled with tap water or a water tank [4] (Fig. 1).

To optimize images, it is crucial to adjust gain, frequency, and focal zones [5]. Comparison to the contralateral part in a dual image is important to differentiate normal from abnormal structures. For vascular structures, we can use a graded compression technique and Doppler functions to access the patency and flow status within the vessels. Dynamic examination is a hallmark of musculoskeletal ultrasound and can be used to evaluate joints and integrity of the muscles and tendons (Additional file 1: Video 1).

## Sonoanatomy

Three hyperechoic and continuous structures can be viewed as sonoanatomy landmarks for the soft tissue and musculoskeletal structures in the extremities. The superficial layer is the skin and dermis, the middle layer is the fascia, and the deepest layer is the cortical surface of the bone. The cortical surface can be further confirmed by rotating the transducer to create a hyperechoic surface with an acoustic shadow (Additional file 2: Video 2).

The subcutaneous tissue is located between the skin and fascia and composed with anechoic fat and discrete hyperechoic connective tissues. Most of the soft tissue infections can take place in this part.

The muscles are located beneath the fascia. Muscle fibers are elongated structures and wrapped up by an outer hyperechoic epimysium. The muscle elements are hypoechoic and surrounded by the echogenic connective tissue. Muscles appear as a spindle appearance in longitudinal scans and a speckled appearance in transverse scans.

The tendons, ligaments, and nerves have similar sonoanatomy: an echogenic, fibrillar pattern in long-axis scans and an echogenic, speckled pattern in transverse-axis scans. The neural elements are hypoechoic, and the surrounding connective tissues are echogenic. We can trace the nerve along its route from the distal to proximal part.

The tendons, ligaments, muscles, and nerves all have anisotropy and should be examined perpendicularly to avoid misinterpreting artifacts as abnormal pathologies.

The vessels have an anechoic, oval, or round pattern in transverse-axis scans and an anechoic, tubular pattern in long-axis scans. In contrast to the veins, the arteries have a thicker, hyperechoic, and consistent wall with a pulsatile nature.

The bones are highly echogenic on the cortical surface and have an acoustic shadow on transverse-axis scans. Disruption of the continuous cortical surface is the hallmark for fracture diagnosis.

## Soft tissue infection

Cellulitis is the most common type of soft tissue infection and confined within the subcutaneous compartment. Cellulitis is a clinical diagnosis. Patients may have fever, chills, and leukocytosis in addition to redness, swelling, local heat, and swelling on the infected sites. A sonographic cobblestone-like appearance is composed of a hyperechoic, hyperemic pattern of the inflamed subcutaneous fat intersecting by anechoic fluid along the connective tissue (Additional file 3: Video 3). However, a cobblestone-like appearance only indicates inflamed tissue and is nonspecific for cellulitis.

**Fig. 2:**
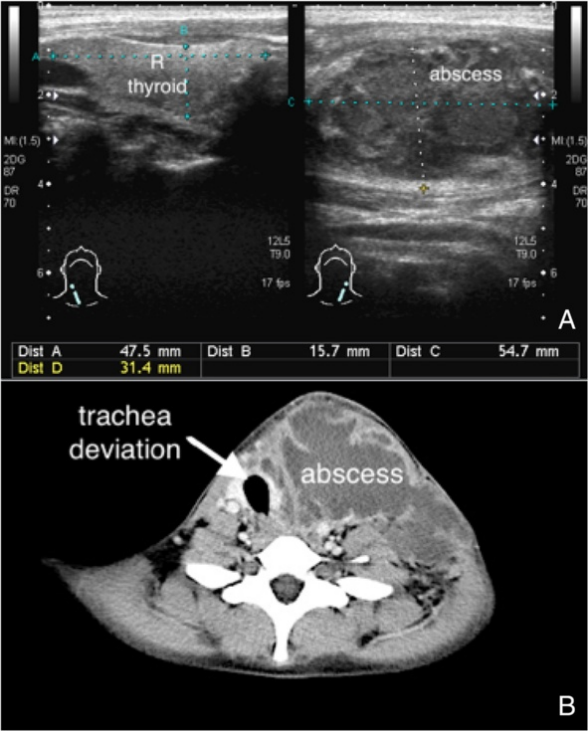
Neck abscess. Left neck abscess had a mixed echogenic content (**a**) and deviated the trachea to the right side (**b**)

The value of ultrasound is to identify occult abscess. POCUS has been shown to alter patient management in up to half patients with abscess [6]. POCUS can also improve the diagnostic accuracy of soft tissue infection in pediatric patients [7]. Abscess is a more severe form of soft tissue infection and has various and possible mixed types of internal echogenicity surrounding the inflamed and thickened subcutaneous tissue (Additional file 4: Video 4). POCUS can be used to diagnose occult abscess, decide the safe route for abscess incision or drainage, and avoid complications during abscess evacuation in either a static or dynamic manner [8, 9]. Squish sign is a movement of echogenic particles in response to compression and can be used to differentiate abscess from soft tissue mass (Fig. 2, Additional file 5: Video 5). It is important to apply Doppler functions to differentiate a pseudoaneurysm from an anechoic abscess.

**Fig. 3:**
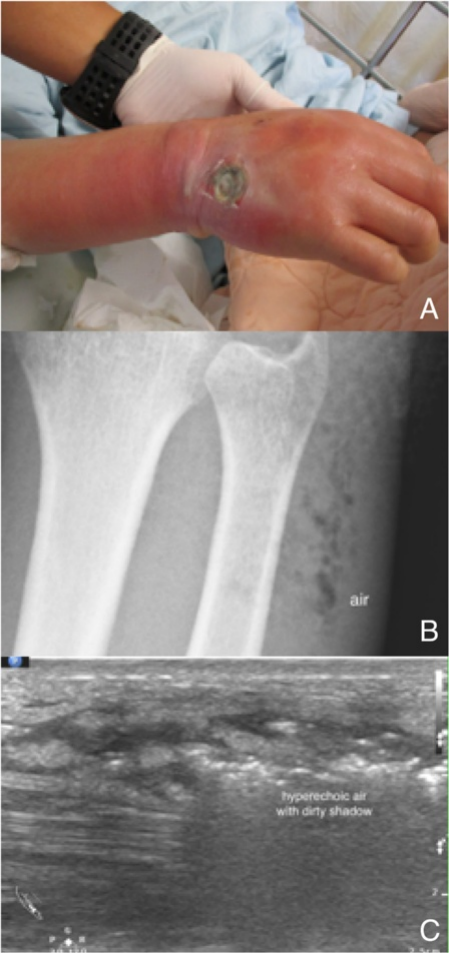
Necrotizing fasciitis with soft tissue gas. A middle-aged man with poorly controlled diabetes mellitus had a severe soft tissue infection on his right hand and forearm (**a**). Soft tissue air was obvious on his forearm radiography (**b**) and ultrasound image (**c**)

Necrotizing fasciitis is a life-threatening soft tissue infection and accompanied by a rapid disease progression and clinical deterioration. Rapid diagnosis is crucial for rapid treatment and better prognosis. The laboratory risk indicator for necrotizing fasciitis (LRINEC) score is a diagnostic scoring system for assessing the severity of soft tissue infection. EPs should consider the presence of necrotizing fasciitis in patients with a LRINEC score for more than or equal 6 [10]. The computed tomography has great value for deeper structure infection but limited value on superficial infection. The magnetic resonance imaging is a great modality for soft tissue infection but is not an adequate imaging modality for those critically ill patients with unstable hemodynamics. POCUS with a linear transducer has been shown to diagnose necrotizing fasciitis with great accuracy in the emergency department [11]. The sonographic features of necrotizing fasciitis are a thickening of subcutaneous tissue and a continuous fluid accumulation for more than 4 mm on the fascia layer with or without comet-tail appearance of gas within infected soft tissues [12] (Fig. 3, Additional file 6: Video 6). In one case report, POCUS had been shown to be a better diagnostic modality compared to computed tomography and magnetic resonance imaging [13].

## Joint effusion

Tender and swollen joints are common in the emergency departments. Different types of arthritis and injuries around joints are often complicated with joint effusions. Bursitis and arthritis with joint effusions are often difficult to differentiate at first encounter. Bursitis is the inflammation of a bursa and accompanied by fluid within the bursa. The joint effusion is located within the joint cavity and has different appearance on a dynamic examination.

**Fig. 4:**
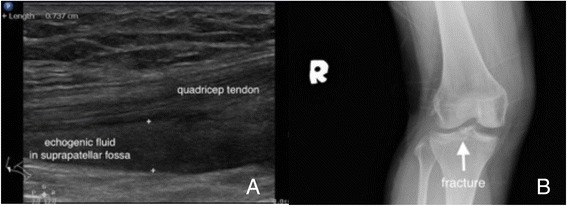
Hemarthrosis. A woman fell and landed on her right knee. Sonographic examination on her swollen knee revealed an echogenic effusion within the suprapatellar fossa (**a**). An X-ray on her knee revealed a tibial plateau fracture (**b**)

Joint effusions developed after injuries usually implicate severe insults to surrounding structures, such as injuries to ligaments, cartilages, and even occult fractures [14, 15]. Joint splinting and advanced imaging modalities should be considered for those injuries complicated with joint effusions (Fig. 4, Additional file 7: Video 7).

POCUS is ideal for inflamed or fluid structures diagnosis. POCUS had changed the management in 65 % of patients with joint pain, erythema, and swelling and reduced the planned joint aspiration from 72.2 to 37 % [16]. Early arthrocentesis can lead to early diagnosis and symptom relief [17]. EPs can use POCUS to guide arthrocentesis in a safer and shortest route to reduce failed attempts and complications. EPs can also use POCUS to avoid blind needling into inflamed structures. Pediatric emergency physicians have been shown to use POCUS to diagnose hip effusion in pediatric patients in the emergency department [18]. Operators can perform POCUS-assisted arthrocentesis in a static or dynamic manner based on their experiences and preferences. Operators can directly visualize the needling during the whole procedure. However, the dynamic POCUS-assisted arthrocentesis requires more practice and a sterile transducer covering for the procedure (Additional file 8: Video 8).

## Foreign bodies

Foreign body-related injuries are often missing on the first encounter and the major reasons for malpractice. Radiopaque foreign bodies in soft tissues and muscles are easily identified by traditional radiography. EPs can use ultrasound as the initial screening modality to identify radiolucent foreign bodies to prevent wound complications and malpractice claims [19, 20]. In one experimental study, emergency doctors identified 29 of 30 foreign bodies and emergency trainees identified 60 of 70 foreign bodies [21]. In one experimental study, nurse practitioners detected 47 of 60 foreign bodies after 2-h POCUS training session [22].

**Fig. 5:**
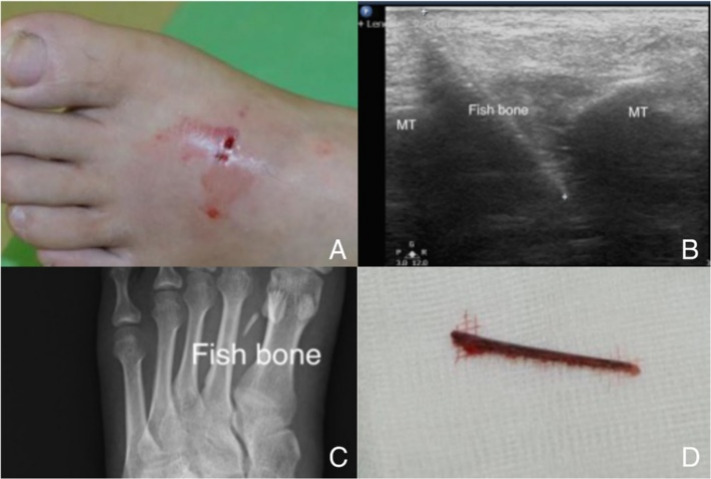
A fish bone in the foot. A middle-aged man accidentally punctured his foot with a fish bone (**a**). The fish bone punctured deeply between the metatarsal bones (**b**). The length of the fish bone was underestimated on radiography (**c**) compared to ultrasound measurement and its actual length (**d**)

The echo patterns for foreign bodies depend on the nature, size, and retention time of the embedded materials. Inflammation around the foreign body becomes obvious for those with longer retention time and creates a surrounding hypoechoic “halo ring”. The surfaces of foreign bodies are always hyperechoic [23]. Different sizes and natures for the foreign bodies can have various artifacts, such as acoustic shadows, comet-tail artifacts, and reverberation artifacts. For open wounds, the transparent Tegaderm can be use to cover the wound prior to scan. For the fingers and toes, we can use water tank to create an ideal window to observe the foreign bodies just beneath the skin and dermis (Fig. 5).

In addition to diagnose foreign bodies, POCUS can be used to assist foreign body retrieval [24, 25]. EPs can use POCUS-assisted in-plane needling to target the foreign body on both sides of the transducer and then explore the target under the needle guidance in traditional manners.

## Long bone fracture

**Fig. 6:**
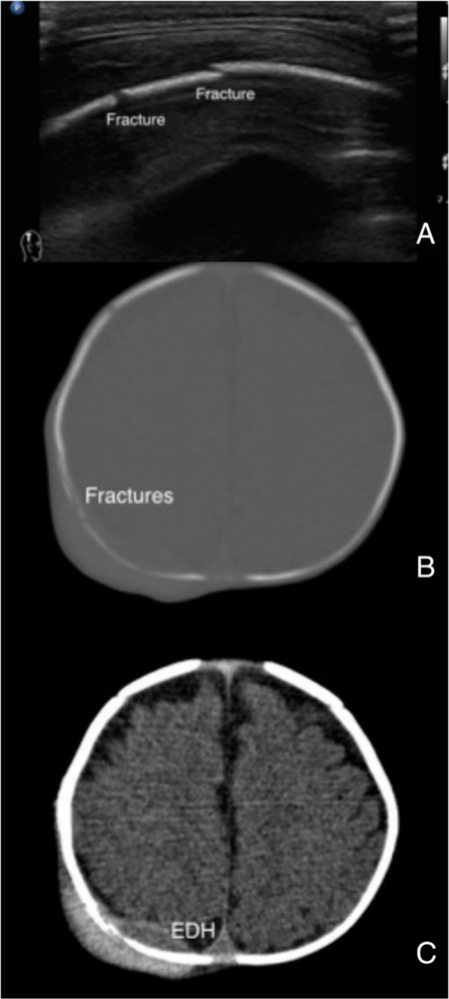
Skull bone fractures. A 3-month-age boy accidentally fell on the ground and developed right occipital hematoma. Fractures were noted under sonographic scans (**a**) and confirmed by the computed tomography (**b**). Small epidural hematoma was noted at the fracture sites (**c**)

POCUS has been shown to be accurate for long bone fracture diagnosis [26, 27]. The step-off sign of the hyperechoic cortical surface is the characteristic finding for fracture diagnosis. Hematoma and soft tissue swelling can be observed as indirect evidence around the fracture sites. The principle for fracture diagnosis is to apply the transducers on the swollen or the most painful regions. We start from transverse-axis scans to define the depth of the target and then rotate to long-axis scans to confirm the fracture based on disruption of the continuous, hyperechoic cortical surface (Additional file 9: Video 9). The principle can be applied to the bones with smooth surface, such as the skull bone and patella (Fig. 6, Additional file 10: Video 10).

**Fig. 7:**
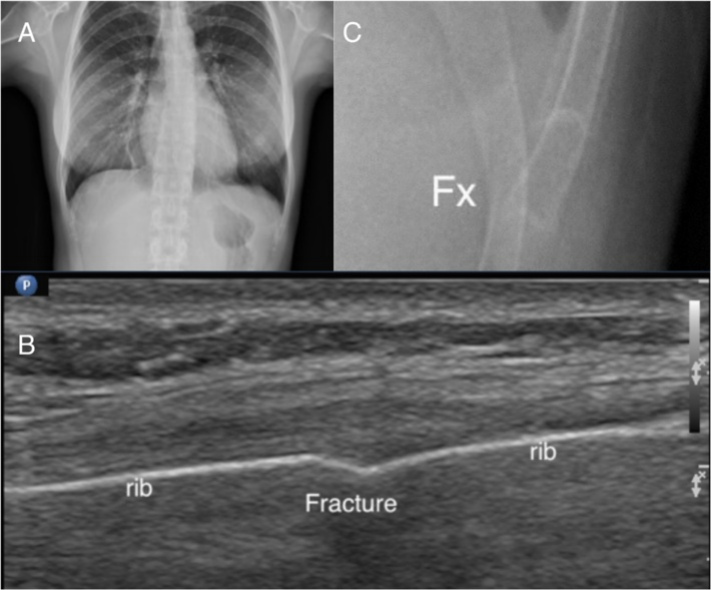
Occult rib fracture. A middle-aged woman suffered from left lower chest contusion in a traffic collision. No obvious fracture was found on initial chest X-ray (**a**). Focused ultrasound on the tender point revealed cortical disruption on the three rib bones (**b**). Enlarged X-ray can only reveal one equivocal rib fracture (**c**)

**Fig. 8:**
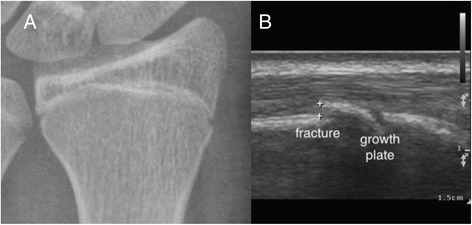
Occult forearm fracture. A teenage boy injured his wrist after a fall. Initial X-ray of the wrist revealed no fracture or soft tissue swelling (**a**). Focused ultrasound with a linear transducer revealed a small fracture close to the growth plate (**b**)

**Fig. 9:**
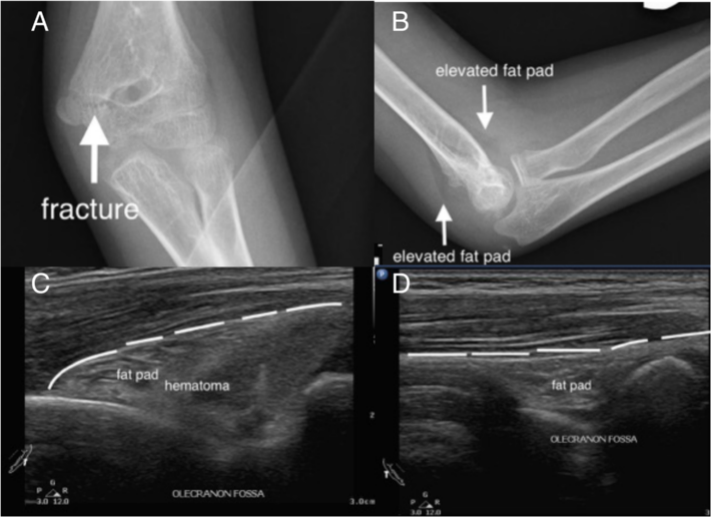
Sonographic fat pad sign. A teenage boy injured his elbow after a fall. The X-ray showed a radiolucent fracture line and elevated fat pad signs (**a**, **b**). Focused ultrasound on the olecranon fossa revealed the elevated fat pad and a hematoma (**c**). The normal fat pad has a smooth margin (**d**)

For the bones with irregular shapes or ends, POCUS for fracture diagnosis is not as accurate as shaft parts and should not be used as the only imaging modality. POCUS can provide valuable information for suspicious occult fractures, such as the ribs (Fig. 7) and fractures near the growth plates (Fig. 8). For fractures close to joints, POCUS is an ideal tool to seek for signs of occult fractures, such as elevated fat pad and associated hematoma [28] (Fig. 9). Hemarthrosis is a hint for intraarticular injuries and can be easily observed by POCUS [14, 15]. POCUS can be used to assist fracture reduction, determine realignment, and perform hematoma block [29–31]. In mass casualty incidents, POCUS for long bone fractures has been integrated in the ultrasound-assisted chest, abdomen, vena cava, and extremities for acute triage (CAVEAT) examination for triage [32].

## Muscle and tendon injuries

Muscle and tendon strains are not POCUS applications. However, POCUS can provide valuable information for major muscle tear and intramuscular hemorrhage. Major muscle tear may appear as irregular and disruption of fibrillar echo texture of muscle bundles and surround mixed echogenicity of hematoma. Dynamic examination of the injured muscle and comparison to the contralateral part can facilitate the diagnosis (Additional file 11: Video 11). Forceful coughing, direct trauma, or coagulopathy can cause intramuscular hemorrhage [33]. The intramuscular hemorrhage can have mixed echogenicity, and Doppler function can aid the diagnosis for pseudoaneurysm formation (Additional file 12: Video 12).

Major tendons, such as Achilles tendon, quadriceps tendon, and patellar tendon, are superficial structures and hence the ideal targets of POCUS [34–36]. EPs can use POCUS to assess the integrity of the fibrillar echo texture of the tendons. Disruption of the typical fibrillar patterns of tendons under dynamic examination is the characteristic finding of tendon ruptures (Additional file 1: Video 1).

## Vascular occlusion

**Fig. 10:**
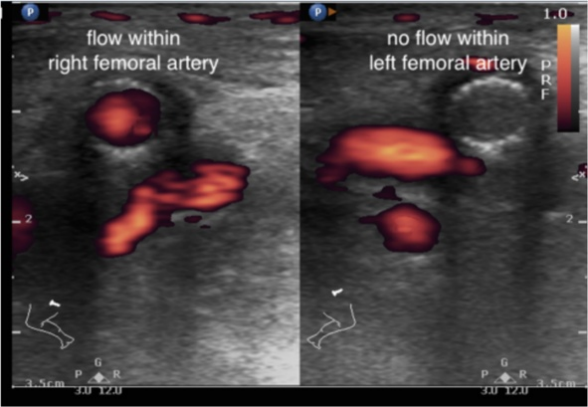
Femoral artery occlusion. An old man has a history of bilateral peripheral artery occlusion disease and been treated with bilateral femoral artery stenting. He developed severe pain on his left leg and found a cold and cyanotic leg and foot. POCUS with power Doppler function revealed no blood flow signals within his left stenting femoral artery

Vascular occlusion, except superficial veins, often causes serious ischemic complications. Deep vein thrombosis is one of core applications EPs should acquire [1]. Familiar with the vascular distribution can lead to rapid targets identification. Unable to completely compress the vein should raise the suspicion for venous thrombosis [37] (Additional file 13: Video 13). Doppler function is mandatory for arterial thrombosis diagnosis (Fig. 10). We suggest following steps for vascular examination: perform transverse-axis scans on target vessels, compress the vessels to differentiate arteries from veins, and rotate the transducer for longitudinal-axis scans with Doppler examinations. There are several methods to reduce mistakes and pitfalls for novice-physicians performed limited compression vascular test: integrating Wells score and limited-compression ultrasound, adding femoral and deep femoral veins as scanning targets and better accessing to the popliteal veins [38–41].

## Procedures

EPs use various emergency procedures for different purposes, such as diagnosis, treatment, monitoring, and resuscitation. Most EPs learn and perform procedures by identifying anatomic landmarks. Novice providers have more failure attempts and higher complications compared to experienced providers. For challenging cases, even experienced hands can encounter obstacles [42]. With the aids of POCUS, EPs can visualize the targets and provide accurate critical procedures instead of anatomic or blind techniques [43, 44].

Echo-guided procedures can be static or dynamic. In static way, EPs use ultrasound guidance to identify the target and then perform the procedures in usual manner. In dynamic way, EPs use non-dominant hand to locate the target and then use dominant hand to insert the needle and advance the needle under real-time ultrasound guidance. The dynamic way is more accurate than static way by direct needling visualization, but required more psychomotor skill practices and aseptic preparations for the transducer. For novice providers, the static echo-guidance is easy and useful. With more practices, the real-time echo guidance should be the better choice for most invasive procedures.

Echo-guided needle advance can be off-plane or in-plane. Off-plane method is easy for novice providers, but to find the needle tip is the hardest part. To identify the needle tip, providers can gradually use tilting method to identify the strong echo of the needle tip and then decide whether to advance the needle or not. Providers can advance the needle and see the entire needle under in-plane ultrasound guidance. The hardest part for in-plane method is to put the needle in the middle part of the transducer throughout the procedures. The needle can be invisible if providers advance the needle in a skewed way.

**Fig. 11:**
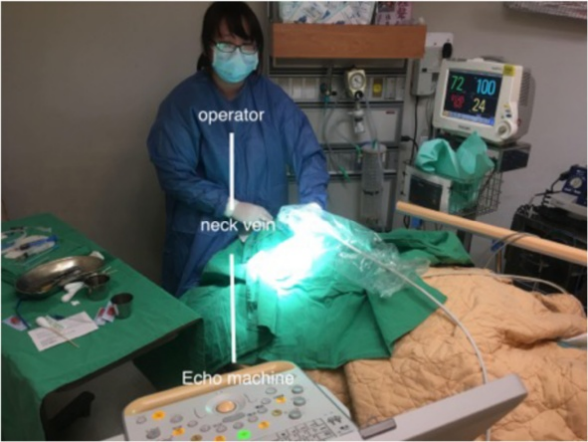
Dynamic echo-guided central venous catheterization. Note the in-line relationship between the operator, patient’s neck vein, and the echo machine

**Fig. 12:**
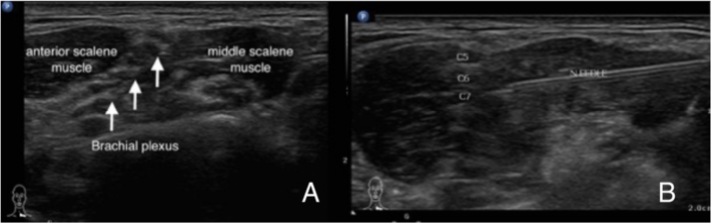
Echo-guided interscalene brachial plexus block for shoulder reduction. **a** The brachial plexus between the anterior and middle scalene muscles. **b** Dynamic in-plane needle approaching to the left brachial plexus

Echo-guided central venous access has been listed as one of high-quality performance. A recent review indicates the benefit of ultrasound-guided central venous catheterization: increase success at first attempt, fewer complications, and less time for cannulation [44]. Providers can perform either off-plane or in-plane guidance according to their preferences. Providers should at least use ultrasound to locate the target central vein. Under adequate antiseptic preparation and covering, dynamic echo-guided catheterization should be the better technique. EPs can use POCUS to assist venous access, to identify the catheter after the procedure and to monitor the complications, such as local hematoma or pneumothorax (Fig. 11, Additional file 14: Video 14). The same principles applied to echo-guided peripheral venous access [45]. EPs can also use POCUS to assist lumbar punctures for patients without adequate anatomy spinal landmarks [46]. Recently, ultrasound-guided nerve blocks have been the emerging field for acute pain management and painful procedures in the EDs [47–49] (Fig. 12, Additional file 15: Video 15). Commercial phantoms or gelatin products can be used to practice and master needling skills [50].

## Conclusions

The skin, soft tissue, and most parts of the musculoskeletal system are relatively the superficial anatomical structures and ideal targets for ultrasound examination. Familiar with sonoanatomy of different structures and psychomotor skills for needling are essential for various ultrasound applications. With POCUS, EPs can visualize the structures beneath the skin and provide better and safer cares in the emergency departments.

## Abbreviations

EP, emergency physician; POCUS, point-of-care ultrasound

## Additional files

Additional file 1: Video 1. Dynamic examination for Achilles tendon rupture. (MP4 6.01 mb) Additional file 2: Video 2. The right thigh landmarks: the skin, fascia, and femur. (MP4 2.77 mb) Additional file 3: Video 3. Cobblestone-like appearance of leg cellulitis. (MP4 3.54 mb) Additional file 4: Video 4. Buttock abscess. (MP4 2.96 mb) Additional file 5: Video 5. Squish sign with a mobile content on compression. (MP4 441 kb) Additional file 6: Video 6. Necrotizing fasciitis with a thickened subcutaneous tissue, an accumulation of fluid, and air within the soft tissue. (MP4 1.61 mb) Additional file 7: Video 7. Hemarthrosis. A longitudinal scan on the right suprapatellar region. (MP4 1.38 mb) Additional file 8: Video 8. Dynamic arthrocentesis on the right elbow. (MP4 5.35 mb) Additional file 9: Video 9. Normal sternum. (MP4 3.16 mb) Additional file 10: Video 10. Skull bone fractures. (MP4 9.38 mb) Additional file 11: Video 11. Left gastrocnemius muscle tear. (MP4 17.0 mb) Additional file 12: Video 12. Rectus muscle hematoma. (MP4 6.23 mb) Additional file 13: Video 13. Left femoral vein thrombosis. (MP4 9.20 mb) Additional file 14: Video 14. Dynamic echo-guided central venous catheterization. (MP4 12.0 mb) Additional file 15: Video 15. Interscalene brachial plexus block. (MP4 2.46 mb)
